# Dysregulation of Iron Metabolism in Cholangiocarcinoma Stem-like Cells

**DOI:** 10.1038/s41598-017-17804-1

**Published:** 2017-12-15

**Authors:** Chiara Raggi, Elena Gammella, Margherita Correnti, Paolo Buratti, Elisa Forti, Jesper B Andersen, Gianfranco Alpini, Shannon Glaser, Domenico Alvaro, Pietro Invernizzi, Gaetano Cairo, Stefania Recalcati

**Affiliations:** 1 Center for Autoimmune Liver Diseases, Humanitas Clinical and Research Center, Rozzano, Italy; 2 Dipartimento Medicina Sperimentale e Clinica, University of Firence, Firenze, Italy; 3 Department of Biomedical Sciences for Health, University of Milan, Milano, Italy; 4 Biotech Research and Innovation Centre, Department of Health and Medical Sciences University of Copenhagen, Copenhagen, Denmark; 5 Research, Central Texas Veterans Health Care System, Baylor Scott & White Digestive Disease Research Center, Scott & White Health, Department of Medicine, Texas A&M Health Science Center, Temple, TX USA; 6 Department of Internal Medicine and Medical Specialties, Sapienza University of Rome, Rome, Italy; 7 Division of Gastroenterology and Program for Autoimmune Liver Diseases, International Center for Digestive Health, Department of Medicine and Surgery, University of Milan-Bicocca, Monza, Italy

## Abstract

Cholangiocarcinoma (CCA) is a devastating liver tumour arising from malignant transformation of bile duct epithelial cells. Cancer stem cells (CSC) are a subset of tumour cells endowed with stem-like properties, which play a role in tumour initiation, recurrence and metastasis. In appropriate conditions, CSC form 3D spheres (SPH), which retain stem-like tumour-initiating features. Here, we found different expression of iron proteins indicating increased iron content, oxidative stress and higher expression of CSC markers in CCA-SPH compared to tumour cells growing as monolayers. Exposure to the iron chelator desferrioxamine decreased SPH forming efficiency and the expression of CSC markers and stem-like genes, whereas iron had an opposite effect. Microarray profiles in CCA samples (n = 104) showed decreased H ferritin, hepcidin and ferroportin expression in tumours respect to surrounding liver, whereas transferrin receptor was up-regulated. Moreover, we found a trend toward poorer outcome in CCA patients with elevated expression of ferritin and hepcidin, two major proteins of iron metabolism. These findings, which represent the first evidence of a role for iron in the stem cell compartment as a novel metabolic factor involved in CCA growth, may have implications for a better therapeutic approach.

## Introduction

Cholangiocarcinoma (CCA), a rare and devastating adenocarcinoma arising from malignant transformation of bile duct epithelial cells, is the second most common form of primary liver tumour with increasing global incidence and mortality rates^1^. CCA severity and the limited benefit of the current therapeutic strategies have rendered this disease a major clinical challenge^1,2^. Therefore, understanding the cellular mechanisms underlying CCA cell growth is essential to develop novel chemopreventive and chemotherapeutic strategies.

Novel insights into the process of tumour evolution have been provided by the discovery of cancer stem cells (CSC) in many human solid tumours, including hepatic cancers (reviewed in^3^). CSC are a subset of cells within a tumour endowed with stem-like properties and higher resistance to chemotherapy compared to bulk tumour cells, which are involved in tumour initiation, recurrence and metastasis. CSC can be defined functionally by their extensive capacity to self-renew, express stem cell markers, differentiate into multiple lineages^3^. Moreover, CSC cultured in appropriate conditions resist to anoikis and tend to form 3D tumour spheres (SPH)^4,5^. Although the role of CSC in CCA is still obscure, we have recently highlighted the presence of a stem-like compartment in human liver cancer, including intrahepatic CCA, by using the functional tool of SPH formation^4,6^.

CSC tolerate the stressful conditions present in tumours, such as hypoxia, low pH, oxidative stress, inflammation^7–9^; therefore, the involvement of iron in all of these settings^10^ strongly suggests that disruption of iron homeostasis may play an important role in CSC tumorigenicity and therapeutic resistance.

Iron can contribute to both tumour initiation and progression^11,12^. Excess iron can lead to reactive oxygen species (ROS) formation and mutagenesis, as shown by increased cancer in patients with iron overload, including liver cancer^13^. Moreover, due to their generally elevated proliferative potential, cancer cells have a greater metabolic demand for iron than normal cells and hence express high levels of transferrin receptor (TfR1) to internalize transferrin-bound circulating iron. Indeed, iron chelators exert inhibitory effects on cell growth and have been considered for tumour therapy^11,12^. Over the last years, several studies have shown that reprogramming of iron metabolism is a key function for a tumour cell. In fact, it has been shown that downregulation of both the iron storage protein ferritin and the iron exporter ferroportin (FPN), together with increased TfR1 expression, leads to higher iron availability in a variety of cancer cells resulting in faster cell growth, and adverse prognosis in cancer patients^14–17^. In this study, taking advantage of a recently established and characterized 3D culture model of human CCA-SPH retaining stem-like tumour-initiating features^4^, we evaluated for the first time the role of iron in CCA, particularly focusing on the stem-compartment.

## Results

### CCA stem like cells have a profile of iron retention

**Figure 1:**
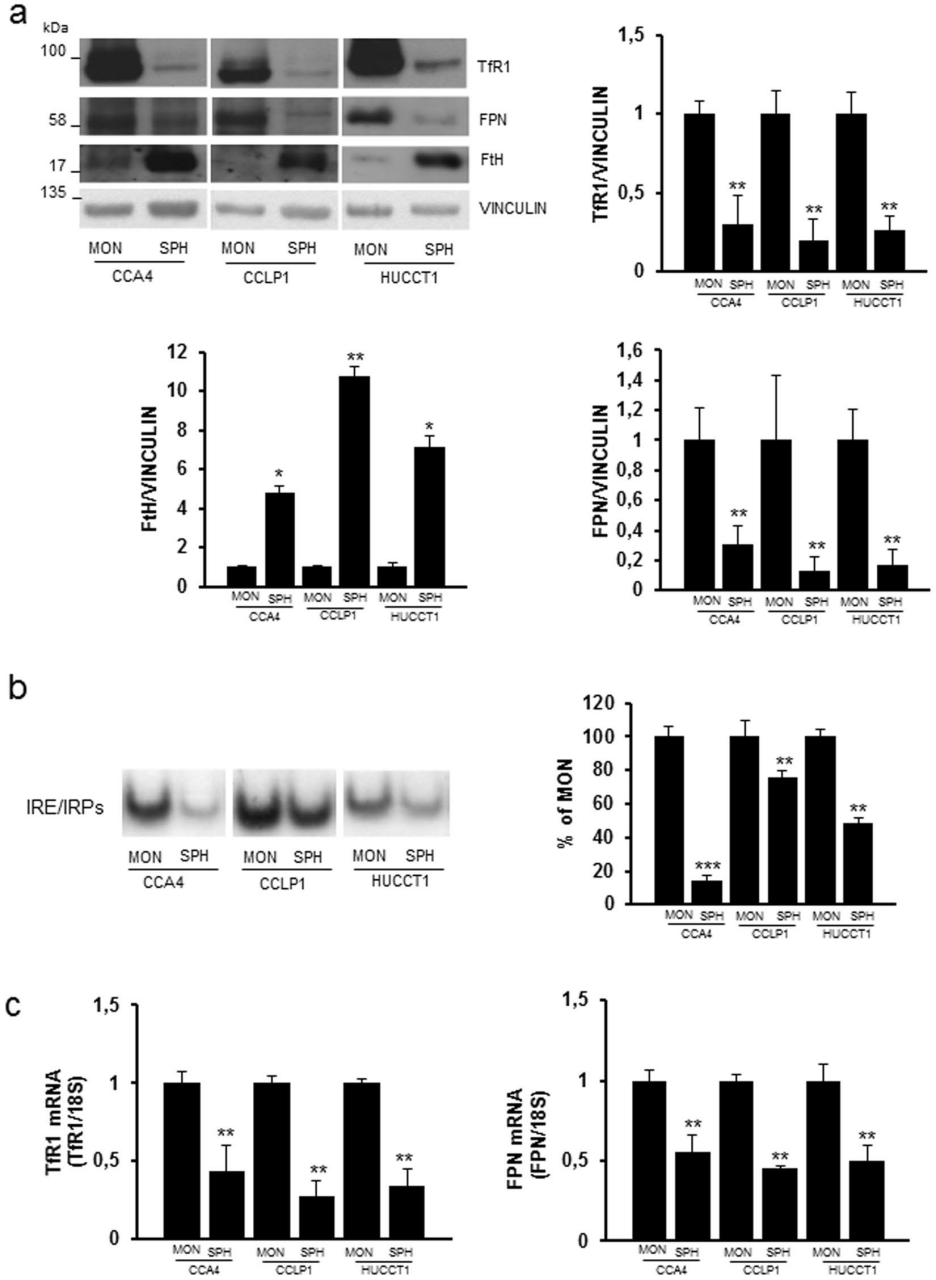
Differential expression of proteins of iron metabolism in CCA cells. The CCA4, CCLP1 and HUCCT1 human CCA-derived cell lines were cultured as adherent monolayers (MON) or in 3D sphere conditions (SPH). Panel a. Top left, representative immunoblot analysis. Cell extracts were reacted with antibodies against transferrin receptor (TfR1), ferroportin (FPN), ferritin H subunit (FtH) and vinculin. Cropped blots are displayed. The original full blot images can be found in Supplementary Information. The graphs show densitometric quantification of immunoblot analyses. The values were normalized to vinculin and expressed as a fraction of respective MON cells normalized to 1. Mean values ± SEM (n = 6), *p ≤ 0.05, **p ≤ 0.01 vs control MON for each cell line. Panel b. RNA bandshift analysis of IRP activity. Cytoplasmic extracts were incubated with a ^32^P-labeled iron-responsive element (IRE) probe and RNA-protein complexes separated on non-denaturing polyacrylamide gels. On the left a representative autoradiogram is shown. A cropped gel is displayed. The original full gel image can be found in Supplementary Information. The graph on the right shows the densitometric quantification of IRPs bands by direct nuclear counting, as described in Materials and Methods; mean percentages ± SEM of control values (n = 6),, **p ≤ 0.01, ***p< 0.001 vs control MON for each cell line. Panel c. TfR1 and FPN mRNA levels were measured by quantitative RT-PCR. Samples were analyzed in triplicate, normalized to the housekeeping gene 18 S and expressed as percentage of respective MON cells normalized to 1. Mean values ± SEM (n = 6), **p ≤ 0.01 vs control MON for each cell line.

We analyzed the expression of the major iron-related proteins in three distinct human intrahepatic CCA cell lines (CCA4, CCLP1 and HUCCT1)^6^ cultured both as adherent monolayers (MON) and in 3D SPH conditions, a culture system recently characterized and validated as a representation of CCA stem-like cells^6^. TfR1 protein levels in SPH were decreased to 10–20% of the value observed in MON (Fig. 1a). Conversely, the amount of ferritin H subunit was much higher in SPH than in attached cells, in line with the inverse correlation between the levels of ferritin and TfR1^10^. These changes, which were mirrored in the levels of TfR1 mRNA (Fig. 1c), indicated that the SPH were iron rich, as confirmed by the lower binding activity of iron regulatory proteins (IRP), which regulate the expression of proteins of intracellular iron metabolism at the post-transcriptional level according to the labile iron pool (LIP)^18^ (Fig. 1b). H ferritin content appears to be controlled by IRP-mediated translational regulation, as we did not find significant differences in H ferritin mRNA levels between SPH and MON. Moreover, the expression of nuclear receptor coactivator 4 (NCOA4), a protein that targets ferritin to autophagic degradation^19^, was the same in MON and SPH (results not shown). Conversely, IRP activity cannot account for the significant decrease of FPN levels in the SPH (Fig. 1a), as FPN translation is repressed when IRP activity is high^18^. The down-regulation of FPN, which is also transcriptionally regulated^20^, may be the result of the decrease in FPN mRNA in SPH relative to MON (Fig. 1c). These modifications of cellular iron metabolism seem to be related to the switch from MON to SPH rather than to malignant transformation *per se*, as in general iron proteins in normal immortalized cholangiocytes (H69 cell line) were not significantly different than in CCA MON, with the exception of FPN, which was less expressed in tumour cells, in line with previous results in other tumours^11^ (Supplementary Fig. 1). Altogether, these findings indicate that the iron content is higher in SPH than in MON.

### Iron dependency of SPH formation

**Figure 2:**
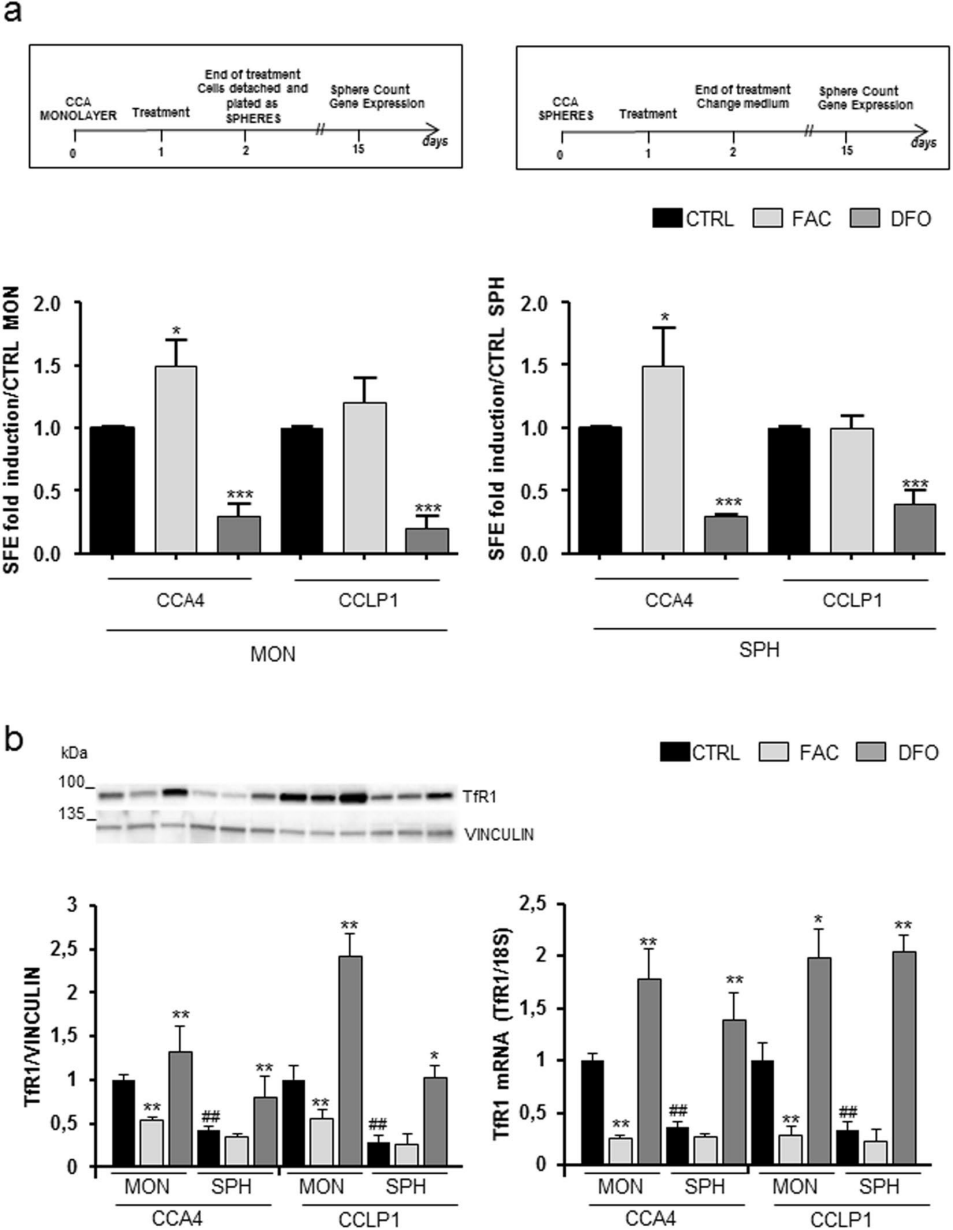
Effect of different iron availability on self-renewal capacity *in vitro*. Panel a. Top: schematic overview of the treatment protocols. Bottom: sphere-forming efficiency (SFE) of CCLP1 and CCA4 MON or SPH untreated (CTRL), treated with ferric ammonium citrate (FAC) (100 µg/ml) or desferioxamine (DFO) (100 µM). Mean ± SEM (n = 3), *p ≤ 0.01, ***p ≤ 0.001 vs CTRL MON (left) or vs CTRL SPH (right) for each cell line. Panel b. TfR1 expression in CCLP1 and CCA4 cell lines cultured and treated as described above. Left: cell extracts were reacted with antibodies against transferrin receptor (TfR1) and vinculin. A representative immunoblot analysis and the densitometric quantification are shown. The values were normalized to vinculin and expressed as a fraction of respective MON cells normalized to 1. Mean values ± SEM (n = 6), *p ≤ 0.01, **p ≤ 0.01 vs CTRL MONO for each cell line; ^##^p ≤ 0.01 vs CTRL SPH. Right: TfR1 mRNA levels were measured by quantitative RT-PCR as described in the legend to Fig. 1. Mean values ± SD (n = 3), *p ≤ 0.05, **p ≤ 0.01 vs CTRL MONO for each cell line.; ^##^p ≤ 0.01 vs CTRL SPH.

To study the functional consequence of this altered expression of iron-related proteins, we evaluated whether changing cellular iron availability by exposure to iron or to an iron chelator may affect sphere-forming efficiency (SFE), as representation of tumour stem compartment^6^. We focused on CCLP1 and CCA4 cell lines, which were treated with ferric ammonium citrate (FAC) or the iron chelator desferrioxamine (DFO) either as MON or SPH according to the experimental design illustrated in Fig. 2a. Treatment with FAC increased SFE significantly only in CCA4 cells, whereas iron chelation showed a greater effect in both cell lines, as the exposure to DFO reduced strongly the SFE in both CCLP1 and CCA4 cells (Fig. 2a). The involvement of iron in SFE was supported by data showing TfR1 expression analyzed in parallel under the same conditions (Fig. 2b). In fact, the analysis of TfR1 at both the mRNA and protein levels, as indicator of intracellular iron levels, showed that the effect of DFO was stronger in SPH, possibly because of their higher basal iron content. Overall, these results indicated that SPH have increased iron dependence.

### Increased ROS production in SPH

**Figure 3:**
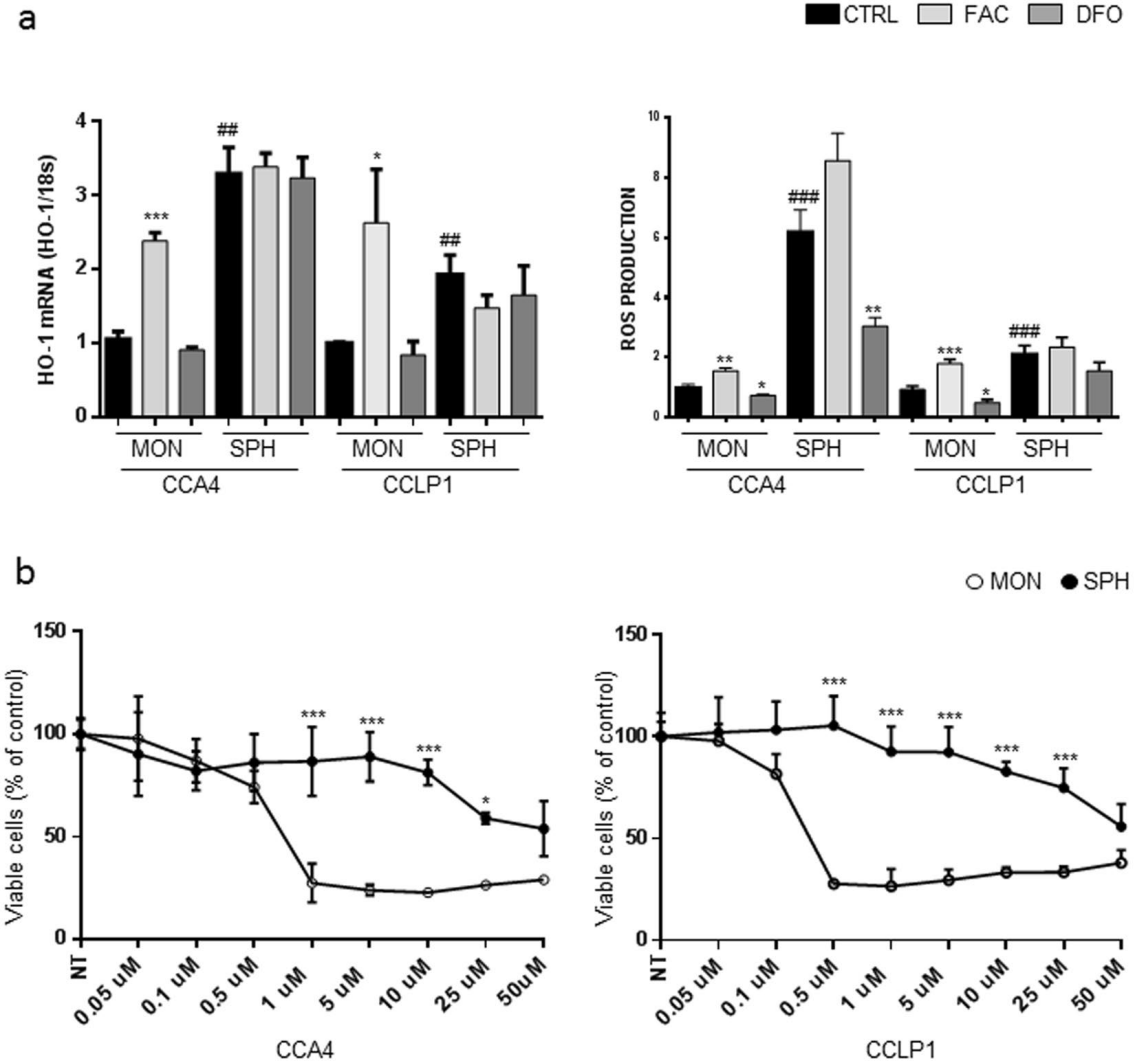
Redox status and ferroptosis in CCA cells growing as monolayers or spheres. Panel a. CCLP1 and CCA4 cells cultured both as MON and SPH were left untreated (CTRL), treated with ferric ammonium citrate (FAC) (100 µg/ml) or desferioxamine (DFO) (100 µM). Left: HO-1 mRNA levels were measured by quantitative RT-PCR as described in the legend to Fig. 1. Mean values ± SEM (n = 3), *p ≤ 0.05, ***p ≤ 0.001 vs CTRL MON, ^##^p ≤ 0.01 vs CTRL SPH. Right: ROS production was evaluated by means of the DCFDA assay as described in Materials and Methods. Samples were analyzed in triplicate, normalized to protein content and expressed as percentage of untreated cells normalized to 1. Mean values ± SEM (n = 3), *p ≤ 0.05, **p ≤ 0.01, ***p ≤ 0.001 vs CTRL MON; ^###^p ≤ 0.001 vs CTRL SPH. Panel b. MON and SPH were treated for 18 h with increasing concentrations of the ferroptosis inducer erastin and cell viability was evaluated by means of the MTT reduction assay. Mean values ± SEM (n = 5), *p ≤ 0.05, ***p ≤ 0.001 vs MON for each point.

The differences in iron levels were accompanied by changes in oxidative stress: both the expression of heme oxygenase (HO-1), a well-known marker of oxidative stress, and ROS levels, were induced by FAC and decreased by DFO. Notably, under basal conditions, oxidative stress was remarkably higher in untreated SPH than in MON (Fig. 3a). To further address the pathogenetic role of the different iron availability, we evaluated cell growth in MON and SPH after manipulation of iron content by FAC or DFO; the number of viable cells was increased by iron supplementation and decreased by chelation in MON, as expected on the basis of previous studies^21^, and also in SPH (Supplementary Fig. S2).

Since excess iron might render SPH more susceptible to ferroptosis, a recently characterized mechanism which causes regulated cell death through iron-dependent ROS production and lipid peroxidation^22^, we treated CCLP1 and CCA4 cells with erastin, a ferroptosis inducing agent. Exposure to increasing concentrations of erastin progressively decreased cell viability, but SPH were remarkably more resistant than MON at concentrations above 0.5 µM (Fig. 3b).

### Effect of iron on the expression of stem like genes

**Figure 4:**
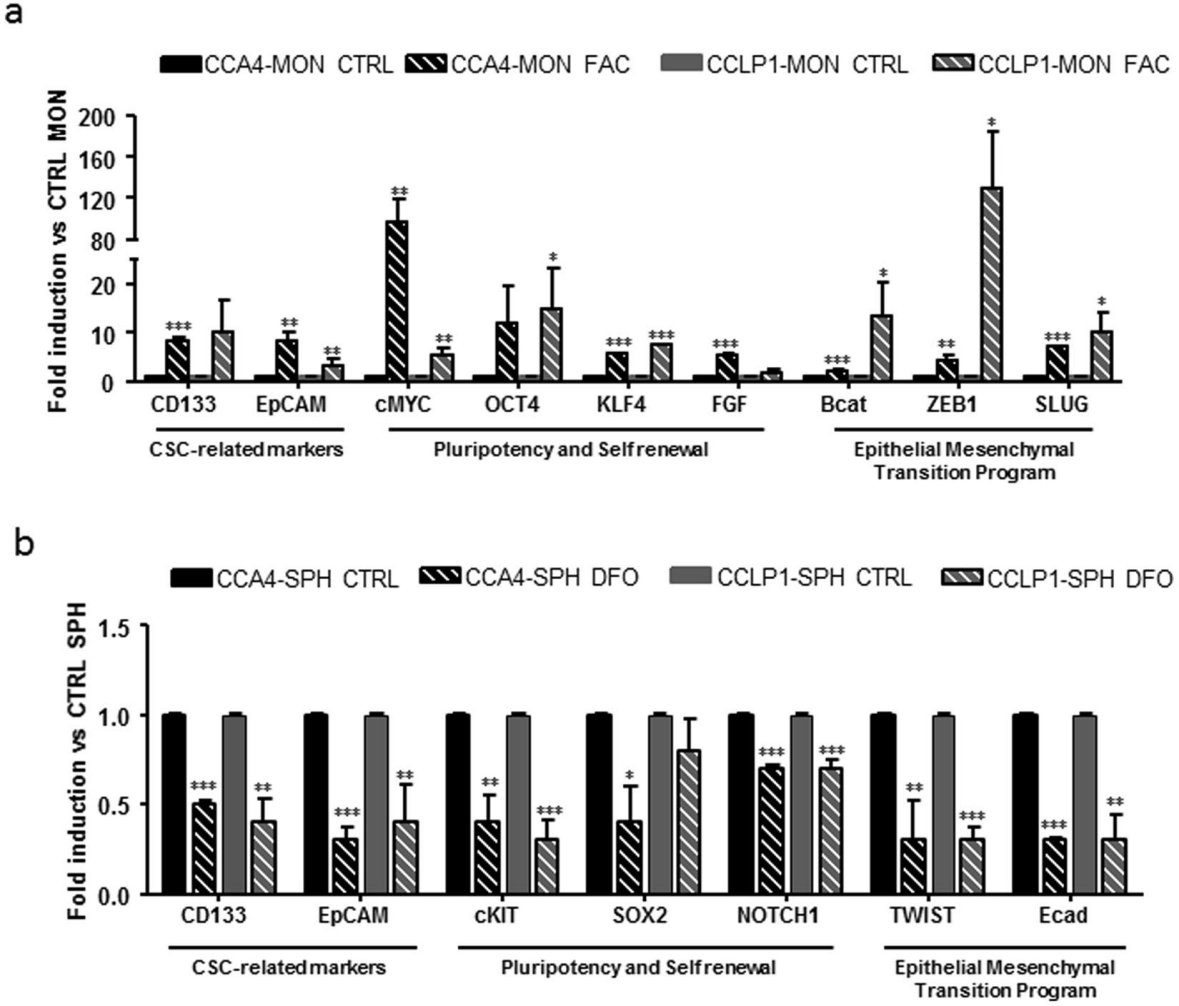
Effect of different iron availability on the expression of stemness-associated genes in monolayers and spheres. CCLP1 and CCA4 cells growing as monolayers (MON) or spheres (SPH) were left untreated (CTRL), treated for 18 h with ferric ammonium citrate (FAC) (100 µg/ml) or with desferioxamine (DFO) (100 µM). The expression of the indicated genes was measured by quantitative RT-PCR as described in the legend to Fig. 1. Panel a. Commonly up-regulated genes in FAC-treated MON. Panel b. Commonly down-regulated genes in DFO-treated SPH. *p ≤ 0.05, **p ≤ 0.01, ***p ≤ 0.001 vs CTRL MON (panel a) or SPH (panel b) for each cell line.

Next, in order to explore the effects of cellular iron levels in the modulation of stem-like genes, we performed a molecular characterization by qRT-PCR of both CCA SPH and MON exposed to DFO or FAC. Overall, MON were more susceptible to FAC treatment (46.2% of genes regulated in CCA4 and 84% in CCLP1) than to DFO (26.9% of genes regulated in CCA4 and 8% in CCLP1), whereas gene expression in the SPH was more affected by iron chelation (84% of genes regulated in CCA4 and 50% in CCLP1 after exposure to DFO) than by iron supplementation (8% of genes regulated in CCA4 and 12.5% in CCLP1). Among a panel of 32 stem-like genes specifically involved in pluripotency and self-renewal, epithelial mesenchymal transition (EMT) and survival, in addition to well-known CSC-related markers, we identified commonly up-regulated genes for both CCA cell lines exposed to iron as MON. In fact, enhanced expression of CD133, epithelial cell adhesion molecule (EpCAM), cMYC, octamer binding transcription factor 4 (OCT4), Kruppel-like factor 4 (KLF4), fibroblast growth factor (FGF), β-catenin, Zinc finger, E-box-binding homeobox 1 (ZEB1) and human Snail family transcriptional repressor (SLUG or SNAI2) genes was found in both CCA4 and CCLP1 MON treated with FAC (Fig. 4a). Similarly, CCA4 and CCLP1 SPH exposed to DFO showed common reduced expression of CD133, EpCAM, tyrosine-protein kinase Kit (cKIT), SRY (sex determining region Y)-box 2 (SOX2), Notch homolog 1 (NOTCH1), Twist-related protein 1 (Twist) and epithelial cadherin (E-cadherin) (Fig. 4b), thus suggesting a clear involvement of iron metabolism in the modulation of tumour stem-like features of CCA.

### Expression of iron related genes in human CCA

**Figure 5:**
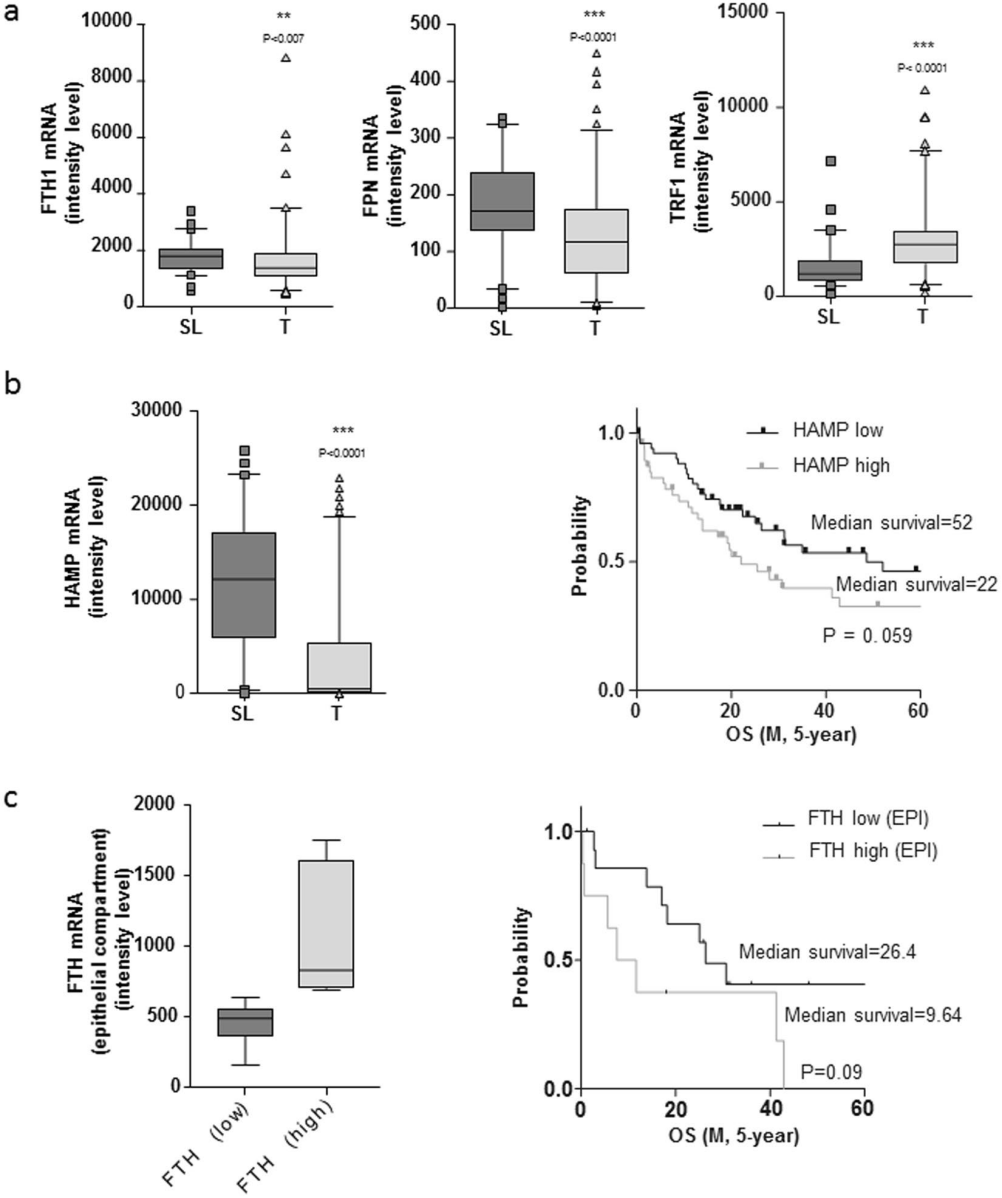
Analysis of correlation between the expression of genes of iron metabolism and CCA patients survival in two different data sets. Panel a. Expression of Ft H subunit (FTH), ferroportin (FPN) and transferrin receptor (TfR1) in CCA tumour samples (T) and healthy liver tissue (SL) in CCA patients. Panel b. Left: expression of hepcidin (HAMP) in CCA tumour samples (T) and healthy liver tissue (SL). Right: correlation between hepcidin expression and survival in a database of 104 CCA patients. Analysis was performed using a Cox proportional hazards model. Overall survival (OS) was demonstrated by Kaplan–Meier and log-rank statistics. Panel c. Left: Ft H subunit gene expression level in intratumoural epithelial (EPI) cells. Right: correlation between Ft H subunit gene expression in the EPI compartment and disease outcome. The epithelial tumour compartment was isolated by laser micro-dissection in a set of CCA patients (n = 23).

To assess whether iron metabolism is altered in CCA, we analyzed the transcriptome of 104 CCA patients^23^. First, we compared gene expression in tumour (T) tissue and matched surrounding liver (SL). H ferritin and FPN mRNA levels in T were significantly reduced compared to SL, whereas TfR1 mRNA levels were up-regulated (Fig. 5a), a result in line with the typical “iron seeking” phenotype previously described in other tumours^11^. We also analyzed hepcidin (HAMP), a liver-derived hormone that controls body iron balance by binding to FPN and inducing its internalization and degradation, thereby blocking iron efflux^24^. HAMP expression was significantly decreased in T compared to SL (Fig. 5b), a result apparently in contrast with the low FPN expression. This strong difference in HAMP expression prompted us to evaluate HAMP expression in CCA cell lines. Compared to the hepatocarcinoma cell line HepG2, HAMP transcript levels were greatly reduced in normal immortalized cholangiocytes (H69 cell line) and decreased further in CCA cell lines (Supplementary Fig. 3), thus explaining the decreased HAMP expression in T. Moreover, HAMP expression did not change significantly between MON and SPH.

### Prognostic relevance of perturbations in iron metabolism in CCA

Next, to study the relationship between iron metabolism and prognosis in CCA, the 104 CCA patients were divided into 2 groups based on their median gene expression level of iron proteins. Correlation analysis with overall survival (OS) showed a trend toward poor prognosis in patients with higher HAMP expression (p = 0.059) (Fig. 5b); analysis of other iron related genes did not show statistical significance. These findings were supported by data obtained analysing The Cancer Genome Atlas (TCGA) database of 38 CCA tumours^25^ in which Kaplan Mayer curves showed that both OS and disease free survival (DFS) were significantly shorter in patients with HAMP gene alterations (amplification or mRNA upregulation) than in subjects without alterations (Supplementary Fig. 4).

Given the high content of stromal cells typically observed in CCA, we then analyzed 23 CCA, randomly selected from the 104 cohort, by laser capture microdissection of the tumour into epithelial (EPI) and stromal cell (S) compartments^23^. Patients with high H ferritin expression in the EPI tumour compartment showed a trend toward shorter OS compared to patients with low H ferritin expression (Fig. 5c). No differences were found in the S compartment.

Taken together these results indicate that patients with higher iron accumulation in tumour cells showed a worse prognosis.

## Discussion

The major finding of this study relates to the mechanisms and consequences of increased iron retention in CCA CSC as a novel metabolic factor involved in CCA growth. We showed that the expression of the major proteins of intracellular iron trafficking is opposite between MON, which have a pattern indicative of low iron content, and SPH, which have a phenotype symptomatic of elevated intracellular iron availability.

Recent evidence showed that the regulation of iron homeostasis is altered in cancer^14–17^; tumour cells generally require more iron than their normal counterparts for a number of functions mainly linked to cell proliferation^21^. Accordingly, most cancer cells show increased expression of TfR1 and reduced levels of FPN, which jointly lead to high intracellular iron availability. Therefore, according to the well-known inverse correlation between ferritin and TfR1^10^, one should expect low ferritin content in tumours, as found in breast cancer cells^11^. However, ferritin upregulation has been reported in several cancer tissues^11^. These apparent discrepancies are possibly explained by increased ferritin expression in stromal^26^ and inflammatory cells^27^ within the tumour. Conversely, the role of ferritin repression in increasing iron availability for the high requirements of tumor cells themselves is supported by studies showing that oncogenes like Myc in B cells^28^, adenovirus E1A^29^ and Ras^30^ downregulate ferritin expression. Inactivation of tumour suppressors may exert a similar effect, as loss of adenomatous polyposis coli (APC) was accompanied by decreased ferritin content in colon cancer^31^, whereas wild type p53 decreases IRP activity^32^ by affecting iron availability^33^ and impairing iron-sulfur cluster assembly^34^, thereby inducing ferritin. In this study, we found a typical pattern (H ferritin low-TfR1 high), indicative of low iron levels, in CCA MON and when we compared tumour tissue with the surrounding healthy liver in CCA patients (Fig. 5a). Conversely, a completely different picture emerged in the SPH, which are representative of CSC, and were characterized by elevated iron content (Fig. 1). Our data strongly suggest that the increased iron level in SPH may result from the low expression of FPN mRNA, which leads to a decrease in FPN levels despite the absence of IRP-mediated translational repression (Fig. 1). Indeed, downregulation of FPN is typical of cancer cells^15,17^, although its role in CSC has not been previously investigated. However, we presently cannot rule out that iron accumulation may be just the consequence of a slower rate of cell division or diminished metabolic consumption.

Notably, the high expression of H ferritin found in SPH is in keeping with the results obtained when we analyzed gene expression selectively in CCA epithelial cells (Fig. 5c), an approach that allowed us to minimize a possible confounding problem related to the great presence of stromal cells in CCA^3^. Although not statistically significant, probably because of relatively small sample number, we found a trend toward shorter survival in patients with higher H ferritin levels, thus suggesting a role for CSC in the CCA of these patients. A similar result was found in glioblastoma stem-like cells^35^ and our findings are also in line with the CSC induction observed in human lung cancer cells exposed to iron^36^. Conversely, a recent study showed that low H ferritin expression is correlated to efficient CCS formation and shorter survival in ovarian cancer^16^. This discrepancy may be related to the several differences existing between distinct types of tumors.

We also found a strong decrease in hepcidin expression in CCA samples as compared to SL, a result in line with the elevated serum hepcidin found in many tumors^11^. Although some cancer cells produce hepcidin^11^, in the case of CCA, hepcidin is probably produced by hepatocytes, as suggested by cell specific differences in hepcidin expression between hepatocytes and cholangiocytes (see Supplementary Fig. 3). However, the shorter survival of patients with high hepcidin expression is in keeping with the inhibitory effect of hepcidin on cellular iron release.

**Figure 6:**
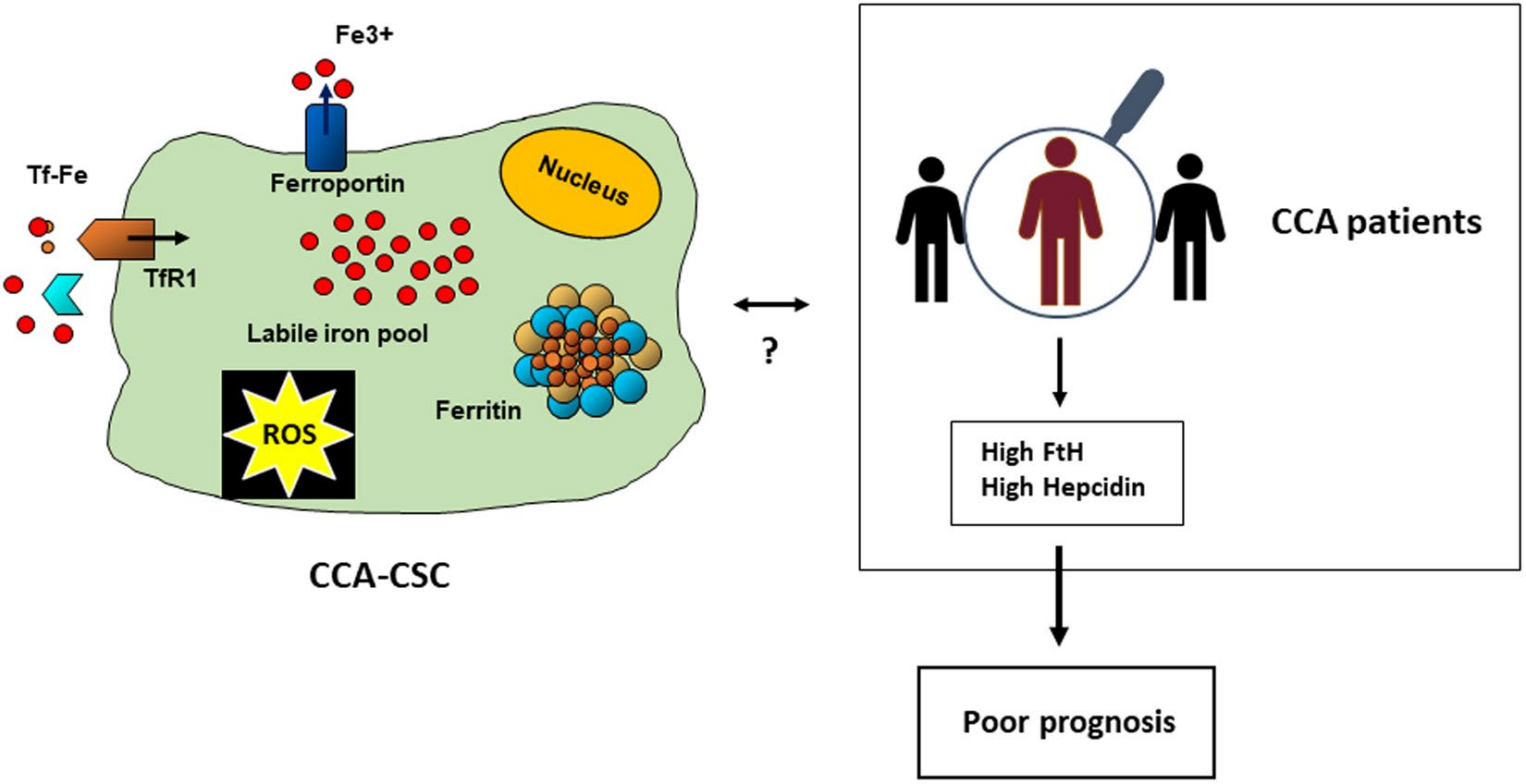
Iron homeostasis is altered in human CCA-CSC and CCA patients. CCA SPH expressing CSC markers are characterized by different expression of iron proteins indicating high iron content and oxidative stress. Likewise, a trend toward poorer outcome was found in CCA patients with high expression of Ft H and hepcidin in cancer cells.

Altogether, these data suggest that the increased iron content in CSC may be a key feature associated with the progression of CCA (Fig. 6).

Whether the increased iron content leads to CSC formation or is a consequence of stemness remains to be determined. In support of the first hypothesis, we found decreased SPH formation in the presence of an iron chelator (Fig. 3), in line with results obtained in lung cancer cells^36^ and medulloblastoma^37^, thus suggesting that the higher amount of iron may have a functional role. In addition, differential gene expression analysis showed that genes involved in stemness and EMT are induced by iron supplementation and down-modulated by iron chelation. In particular, iron modulated the Wnt-β-catenin pathway, as shown by induction of β-catenin and its downstream targets MYC and EpCAM in iron-treated MON and repression in SPH exposed to DFO (Fig. 4). The iron dependence of β-catenin signaling in CCA shown here is in line with the demonstration that several compounds with different structures, but having iron chelating capacity in common, are able to inhibit various types of cancer, such as multiple myeloma^38^ and colorectal cancer^31,39–41^ by targeting β-catenin signalling. This mechanism may be pathogenetically significant, as increased β-catenin levels have been observed in a high percentage of CCA (reviewed in^42^). Furthermore, EMT-inducing transcription factors, such as SLUG and TWIST, which are associated with invasion, poor differentiation and short survival^42^, are also regulated by iron availability (Fig. 5). Moreover, our finding that TfR1 is markedly repressed in SPH, which show high expression of EMT markers relative to MON (Fig. 5), is consistent with the impaired EMT progression found in tumor cells treated with iron chelators, which induce TfR1^43^, and with the recent demonstration that TfR1 inhibits EMT in intestinal epithelial cells^44^. Conversely, glioblastoma stem-like cells, which critically require high levels of ferritin, also showed high expression of TfR1^35^, although the molecular mechanism allowing elevated synthesis of both proteins is not in line with the current model of regulation of intracellular iron homeostasis^10^. In agreement with the connection between elevated iron in the LIP and oxidative stress^10,18^, we found increased ROS levels and induction of HO-1 in SPH compared to MON and we also showed an association between SFE and ROS (Figs 2 and 4). It is well accepted that cancer cells generate more ROS than their normal counterparts, whereas the microenvironment of normal stem cells is characterized by low levels of ROS^45^. On the other hand, much less is known about the biological effects of ROS in CSC, although some studies showed that CSC produce less ROS than other tumour cells and CSC expressing the common marker CD44, which facilitates glutathione synthesis, are more resistant to oxidative stress^46^. We can speculate that elevated ferritin synthesis in CCA SPH is not sufficient to store iron and excessive free iron promotes the formation of radical species. In keeping with the present findings, a similar ROS-mediated induction of CSC was recently found in iron-treated lung cancer cells^36^. The higher levels of iron and ROS in SPH prompted us to investigate whether they were more susceptible to ferroptosis. However, we unexpectedly found that SPH were less vulnerable to ferroptosis, perhaps in relation to their resistance to various types of chemoterapeutic agents^3^.

An alternative hypothesis is that genes associated to CSC may modify iron homeostasis. Since the membrane protein CD133 is a negative regulator of Tf endocytosis, and hence of TfR1-mediated iron uptake^47^, the changes in CD133 expression found in MON and SPH, which are opposite to those of TfR1, may affect CSC iron status and lead to high LIP. Obviously, it is also well plausible that the two possibilities are not mutually exclusive but progressively interact, ultimately disrupting iron homeostasis in CCA. For example, MYC, which plays a key role in liver CSC development and maintenance^48^ and is modulated by cellular iron content (Fig. 4), has been shown to inhibit ferritin expression in tumour cells^28,31^.

The current model of altered iron homeostasis in cancer cells can be summarized in a framework in which increased iron uptake and decreased iron storage and export contribute to enhancing the levels of iron to sustain the high requirement of growing tumour cells. We confirmed this “iron regulatory gene signature”^49^ in MON, but a different picture emerged from the analysis of iron metabolism in CCA SPH, an accepted model of CSC, as our findings revealed elevated iron storage and oxidative stress. This increased iron dependency is accompanied by substantial changes in the expression of CSC markers that can be reversed by iron removal and is mirrored by data indicating a trend toward shorter survival in CCA patients with elevated hepcidin expression and higher H ferritin expression in epithelial cells, indicative of high iron retention (Fig. 6).

By giving insights into the particular regulation of iron homeostasis in the stem compartment of CCA, this study provides advances in the understanding of the pathogenesis of this deadly tumour that may hopefully translate into an effective adjunct therapeutic approach based on iron deprivation.

## Methods

### Cell culture and treatments

The human intrahepatic CCA cell lines CCLP1, HUCCT1 (a kind gift from A.J. Demetris, Pittsburgh) and CCA4 were previously described^6^. The human immortalized non-malignant cholangiocyte cell line H69 was kindly provided by Dr. G. J. Gores (Mayo Clinic, Rochester). The cell lines were grown at 37 °C in a humidified incubator at 5% CO_2_ as monolayers (MON) in DMEM medium or spheres (SPH) in anchoring-independent conditions with selective serum-free DMEM/F12 medium supplemented with 1X B27 supplement minus vitamin A (Life Technologies, Monza, Italy), human recombinant EGF (R&D System, Milano, Italy) (20 ng/mL) and bFGF (R&D System) (20 ng/mL)^4,6^. Cells were treated for 18 hours with 100 µM Desferioxamine (DFO) (Sigma Aldrich, Milano, Italy) or 100 µg/ml ferric ammonium citrate (FAC) (Sigma Aldrich) or increasing concentrations of erastin (Sigma Aldrich).

### Sphere assay

The cells were grown in anchoring-independent conditions (see above^4,6^). After 15 days, the SPH were counted, and the SFE was calculated by dividing the number of SPH by the original number of single cells seeded and expressed as a percentage. SPH morphology was assessed using an Olympus IX81 confocal microscope as previously reported^4^. Large-scale SPH cultures were established by plating 1.8 × 10^3^ cells/cm^2^ into poly (2-hydroxyethyl methacrylate) (poly-HEMA, Sigma Aldrich) coated dishes in the presence of 1% methylcellulose (R&D System).

### Quantitative real-time polymerase chain reaction

Total RNA isolated from SPH or MON using TRI reagent® (Sigma Aldrich) was reverse transcribed into cDNA with Proto Script M-MuLV First Strand cDNA Synthesis Kit (New England Biolabs, Pero, Italy) and the obtained cDNA served as a template for Real-Time PCR. Changes in the mRNA levels were detected by amplification of each sample in triplicate using FAST SYBR-Green PCR Master Mix and the 7900HT Fast Real Time PCR System (Applied Biosystems, Monza, Italy) and gene-specific primer pairs (see Table 1) or by real time PCR based on the TaqMan methodology (Life Technologies) using the primers indicated in Table 2 (Applied Biosystems). The levels of 18 S RNA were used for normalization. For all tested genes, the fold difference (2^−∆∆Ct^) was calculated using the ∆Ct of respective untreated sample as a control. All reactions were performed in triplicate.

**Table 1:** Primers for SYBR-Green PCR.

Gene	Primer Forward	Primer Reverse
**CD133**	GCTTCAGGAGTTTCATGTTGG	GGGGAATGCCTACATCTGG
**EpCAM**	TGTGGTGATAGCAGTTGTTGC	CTATGCATCTCACCCATCTCC
**cKIT**	TTGGGGTTGTGTTGTCACC	TGAAGTGCCCCTGAAGTACC
**cMYC**	CGGAACTCTTGTGCGTAAGG	ACTCAGCCAAGGTTGTGAGG
**OCT4**	TTGTGCCAGGGTTTTTGG	ACTTCACCTTCCCTCCAACC
**KLF4**	AGACAGTCTGTTATGCACTGTGG	TGTTCTGCTTAAGGCATACTTGG
**SOX2**	ATGGGTTCGGTGGTCAAGT	GGAGGAAGAGGTAACCACAGG
**bFGF**	GGCTATGAAGGAAGATGGAAGATT	GGCTATGAAGGAAGATGGAAGATT
**NOTCH1**	GCAGTTGTGCTCCTGAAGAA	CGGGCGGCCAGAAAC
**Bcat**	GCTGGGACCTTGCATAACCTT	ATTTTCACCAGGGCAGGAATG
**TWIST**	GGAGTCCGCAGTCTTACGAG	TCTGGAGGACCTGGTAGAGG
**Ecad**	AGGCCAAGCAGCAGTACATT	ATTCACATCCAGCACATCCA
**ZEB1**	AAGAAAGTGTTACAGATGCAGCTG	CCCTGGTAACACTGTCTGGTC
**SLUG**	GGGGAGAAGCCTTTTTCTTG	TCCTCATGTTTGTGCAGGAG
**Rn18s**	CCACTTTCGATGGTAGTCG	CCTTCCTTGGATGTGGTA

**Table 2:** Primers for TaqMan PCR.

Gene	Primer
**TfR1**	Ms00951083-m1
**Fpn**	Ms00205888-m1
**Rn18s**	Hs03928985-g1
**HO-1**	Ms01110250-m1
**HAMP**	Hs00221783-m1

### Immunoblotting analysis

Proteins in cell extracts were separated by electrophoresis and transferred to nitrocellulose. Filters were incubated for 1 hour at room temperature with the primary antibodies indicated in Table 3 and then incubated with the appropriate horseradish peroxidase-conjugated secondary antibody (Cell Signaling or BioRad, Milano, Italy). The signals were revealed using the ECL method (Amersham, Pero, Italy) and quantified using ImageLab 5.2.1 software with the values being calculated after normalisation to the amount of vinculin.

**Table 3:** Antibodies.

Protein name	Antibody	Incubation
**Fpn**	Novus Biologicals NBP1-21502	1:1500
**FtH**	anti-H Ft subunit monoclonal antibody (see ref.^50^)	1:1000
**TfR1**	Invitrogen 136800	1:500
**Vinculin**	Cell Signalling #4650	1:5000

### RNA-protein bandshift assay

Equal amounts of proteins from cell lysates prepared as previously described^50^ were incubated with a molar excess of IRE probe transcribed *in vitro* from the pSPT-fer plasmid containing the IRE of the human ferritin H chain in the presence of 100 µCi of (α-32P) UTP (800 Ci/mmol) (Perkin Elmer, Milano, Italy) and sequentially treated with RNase T1 and heparin as previously described^50^. After separation on non-denaturing polyacrylamide gels, the IRPs-RNA complexes were visualized autoradiographically and quantified by means of direct nuclear counting using an InstantImager (Packard Instruments, Meriden CT, USA).

### Reactive oxygen species detection

ROS production was monitored using the dichloro-dihydro-fluorescein diacetate (DCFH-DA) fluorescent probe (Sigma Aldrich), a cell-permeable indicator of ROS. Cells cultured as MON or SPH as described above were stained by re-suspending in 20 µM DCFH-DA and incubated at 37 °C for 30 minutes in the dark. Fluorescence was measured (nm: 495 excitation and 529 emission).

### MTT assay

Cells grown as MON or SPH were left untreated or treated with FAC or DFO. At the end of the treatments, cell viability was measured using thiazolyl blue (MTT, Sigma Aldrich, Milano, Italy) as an indicator of mitochondrial function. After incubation with the MTT solution (5 mg/ml) at 37 °C for 2 hours, formazan crystals were dissolved by adding the MTT solubilization solution. Absorbance was read at 570 nm, and the background absorbance at 690 nm was subtracted.

### Statistical analysis

Results are expressed as mean ± SEM, as specified. Statistical significance between groups was assessed by Student’s *t* test with Prism software (version 6.00 for Windows; GraphPad).

### CCA patient data base and statistical analysish

The GSE26566 series matrix containing gene expression values from Illumina humanRef-8 v2.0 bead chips of 104 CCA (T) and 59 matched noncancerous surrounding liver (SL) samples was downloaded from GEO^23^. 23 random CCA cases were selected for laser microdissection of both the tumour epithelial and stromal component. Differences in gene expression of selected genes were evaluated and data represented as box plots with 5–95 percentiles (GraphPad Prism v5). Genes of iron metabolism were also evaluated using The Cancer Genome Atlas (TCGA) database of 38 CCA tumours in which DNA mutations, RNA expression, copy number and DNA methylation were assessed^25^. Statistical significance was calculated using Mann Whitney test (2-tailed). Overall survival was calculated based on the median gene expression and shown as Kaplan-Meier plots with log Rank statistics. Pearson correlation between gene pairs was calculated using R and the “cortest” function, yielding correlation coefficients and p-values.

### Data availability

The datasets generated during and/or analysed during the current study are available from the corresponding author on reasonable request.

## Electronic supplementary material

Supplementary Information

## References

[1] Banales JM (2016). Expert consensus document: Cholangiocarcinoma: current knowledge and future perspectives consensus statement from the European Network for the Study of Cholangiocarcinoma (ENS-CCA). Nat Rev Gastroenterol Hepatol.

[2] Blechacz B, Gores GJ (2008). Cholangiocarcinoma: advances in pathogenesis, diagnosis, and treatment. Hepatology.

[3] Raggi C, Invernizzi P, Andersen JB (2015). Impact of microenvironment and stem-like plasticity in cholangiocarcinoma: molecular networks and biological concepts. J Hepatol.

[4] Raggi C (2014). Epigenetic reprogramming modulates malignant properties of human liver cancer. Hepatology.

[5] Dontu G (2003). *In vitro* propagation and transcriptional profiling of human mammary stem/progenitor cells. Genes Dev.

[6] Raggi C (2016). Cholangiocarcinoma stem-like subset shapes tumor-initiating niche by educating associated macrophages. J Hepatol.

[7] Flavahan WA (2013). Brain tumor initiating cells adapt to restricted nutrition through preferential glucose uptake. Nat Neurosci.

[8] Hjelmeland AB (2011). Acidic stress promotes a glioma stem cell phenotype. Cell Death Differ.

[9] Li Z (2009). Hypoxia-inducible factors regulate tumorigenic capacity of glioma stem cells. Cancer Cell.

[10] Hentze MW, Muckenthaler MU, Galy B, Camaschella C (2010). Two to tango: regulation of Mammalian iron metabolism. Cell.

[11] Torti SV, Torti FM (2013). Iron and cancer: more ore to be mined. Nat Rev Cancer.

[12] Lui GY (2015). Targeting cancer by binding iron: Dissecting cellular signaling pathways. Oncotarget.

[13] Tirnitz-Parker JE, Glanfield A, Olynyk JK, Ramm GA (2013). Iron and hepatic carcinogenesis. Crit Rev Oncog.

[14] Chen Y (2015). Disordered signaling governing ferroportin transcription favors breast cancer growth. Cell Signal.

[15] Tesfay L (2015). Hepcidin regulation in prostate and its disruption in prostate cancer. Cancer Res.

[16] Lobello N (2016). Ferritin heavy chain is a negative regulator of ovarian cancer stem cell expansion and epithelial to mesenchymal transition. Oncotarget.

[17] Pinnix ZK (2010). Ferroportin and iron regulation in breast cancer progression and prognosis. Sci Transl Med.

[18] Recalcati S, Minotti G, Cairo G (2010). Iron regulatory proteins: from molecular mechanisms to drug development. Antioxid Redox Signal.

[19] Mancias JD, Wang X, Gygi SP, Harper JW, Kimmelman AC (2014). Quantitative proteomics identifies NCOA4 as the cargo receptor mediating ferritinophagy. Nature.

[20] Drakesmith H, Nemeth E, Ganz T (2015). Ironing out Ferroportin. Cell Metab.

[21] Yu Y, Kovacevic Z, Richardson DR (2007). Tuning cell cycle regulation with an iron key. Cell Cycle.

[22] Yang WS, Stockwell BR (2016). Ferroptosis: Death by Lipid Peroxidation. Trends Cell Biol.

[23] Andersen JB (2012). Genomic and genetic characterization of cholangiocarcinoma identifies therapeutic targets for tyrosine kinase inhibitors. Gastroenterology.

[24] Ganz T (2011). Hepcidin and iron regulation, 10 years later. Blood.

[25] Farshidfar F (2017). Integrative Genomic Analysis of Cholangiocarcinoma Identifies Distinct IDH-Mutant Molecular Profiles. Cell Rep.

[26] Rossiello R, Carriero MV, Giordano GG (1984). Distribution of ferritin, transferrin and lactoferrin in breast carcinoma tissue. J Clin Pathol.

[27] Alkhateeb AA, Han B, Connor JR (2013). Ferritin stimulates breast cancer cells through an iron-independent mechanism and is localized within tumor-associated macrophages. Breast Cancer Res Treat.

[28] Wu KJ, Polack A, Dalla-Favera R (1999). Coordinated regulation of iron-controlling genes, H-ferritin and IRP2, by c-MYC. Science.

[29] Tsuji Y, Kwak E, Saika T, Torti SV, Torti FM (1993). Preferential repression of the H subunit of ferritin by adenovirus E1A in NIH-3T3 mouse fibroblasts. J Biol Chem.

[30] Kakhlon O, Gruenbaum Y, Cabantchik ZI (2002). Ferritin expression modulates cell cycle dynamics and cell responsiveness to H-ras-induced growth via expansion of the labile iron pool. Biochem J.

[31] Radulescu S (2012). Luminal iron levels govern intestinal tumorigenesis after Apc loss *in vivo*. Cell Rep.

[32] Zhang F, Wang W, Tsuji Y, Torti SV, Torti FM (2008). Post-transcriptional modulation of iron homeostasis during p53-dependent growth arrest. J Biol Chem.

[33] Tong WH (2011). The glycolytic shift in fumarate-hydratase-deficient kidney cancer lowers AMPK levels, increases anabolic propensities and lowers cellular iron levels. Cancer Cell.

[34] Funauchi Y (2015). Regulation of iron homeostasis by the p53-ISCU pathway. Sci Rep.

[35] Schonberg DL (2015). Preferential Iron Trafficking Characterizes Glioblastoma Stem-like Cells. Cancer Cell.

[36] Chanvorachote P, Luanpitpong S (2016). Iron induces cancer stem cells and aggressive phenotypes in human lung cancer cells. Am J Physiol Cell Physiol.

[37] Bisaro B (2015). Proteomic analysis of extracellular vesicles from medullospheres reveals a role for iron in the cancer progression of medulloblastoma. Mol Cell Ther.

[38] Kamihara, Y. *et al*. The iron chelator deferasirox induces apoptosis by targeting oncogenic Pyk2/β-catenin signaling in human multiple myeloma. *Oncotarget*, 10.18632/oncotarget.11830 (2016). 10.18632/oncotarget.11830 PMC5325446 27602957

[39] Brookes MJ (2008). A role for iron in Wnt signalling. Oncogene.

[40] Song S (2011). Wnt inhibitor screen reveals iron dependence of β-catenin signaling in cancers. Cancer Res.

[41] Coombs GS (2012). Modulation of Wnt/β-catenin signaling and proliferation by a ferrous iron chelator with therapeutic efficacy in genetically engineered mouse models of cancer. Oncogene.

[42] Vaquero J (2016). Epithelial-mesenchymal transition in cholangiocarcinoma: From clinical evidence to regulatory networks. J Hepatol.

[43] Chen Z (2012). The iron chelators Dp44mT and DFO inhibit TGF-β-induced epithelial-mesenchymal transition via up-regulation of N-Myc downstream-regulated gene 1 (NDRG1). J Biol Chem.

[44] Chen AC, Donovan A, Ned-Sykes R, Andrews NC (2015). Noncanonical role of transferrin receptor 1 is essential for intestinal homeostasis. Proc Natl Acad Sci USA.

[45] Bigarella CL, Liang R, Ghaffari S (2014). Stem cells and the impact of ROS signaling. Development.

[46] Ishimoto T (2011). CD44 variant regulates redox status in cancer cells by stabilizing the xCT subunit of system xc(-) and thereby promotes tumor growth. Cancer Cell.

[47] Bourseau-Guilmain E, Griveau A, Benoit JP, Garcion E (2011). The importance of the stem cell marker prominin-1/CD133 in the uptake of transferrin and in iron metabolism in human colon cancer Caco-2 cells. PLoS One.

[48] Chow EK, Fan LL, Chen X, Bishop JM (2012). Oncogene-specific formation of chemoresistant murine hepatic cancer stem cells. Hepatology.

[49] Miller LD (2011). An iron regulatory gene signature predicts outcome in breast cancer. Cancer Res.

[50] Cairo G (1997). Inappropriately high iron regulatory protein activity in monocytes of patients with genetic hemochromatosis. Blood.

