# Supplementary material for: Dysregulation of Iron Metabolism in Cholangiocarcinoma Stem-like Cells
Source: Sci Rep. 2017 Dec 15;7:17667. doi: 10.1038/s41598-017-17804-1 (PMC5732280; doi:10.1038/s41598-017-17804-1)

Dysregulation of Iron Metabolism in Cholangiocarcinoma Stem-like Cells

Chiara Raggi, Elena Gammella, Margherita Correnti, Paolo Buratti, Elisa Forti, Jesper B Andersen, Gianfranco Alpini, Shannon Glaser, Domenico Alvaro, Pietro Invernizzi, Gaetano Cairo and Stefania Recalcati

Supplemental figures

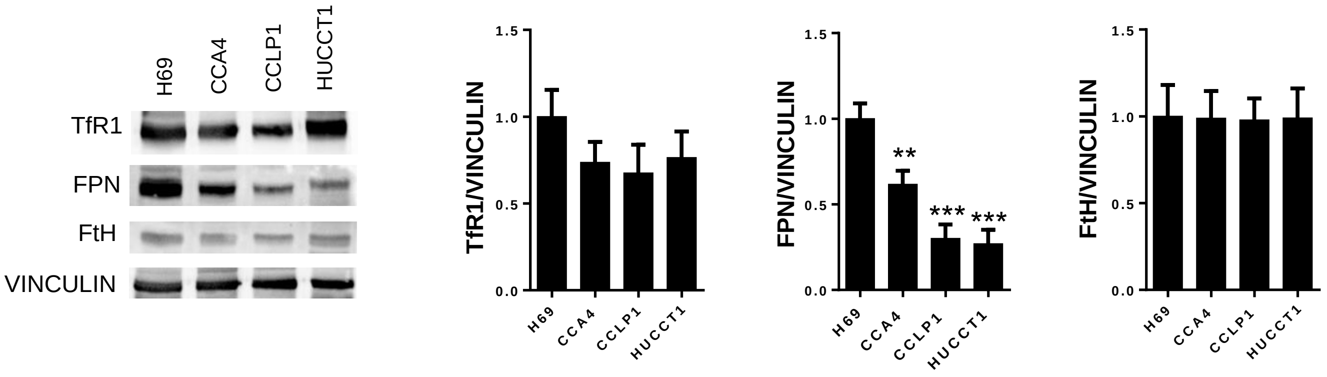

Supplemental Figure 1. Expression of proteins of iron metabolism in CCA cell lines and normal immortalized cholangiocytes

Human CCA-derived cell lines and normal immortalized cholangiocytes (H69 cells) were cultured as adherent monolayers. Representative immunoblot analysis: cell extracts were reacted with antibodies against transferrin receptor (TfR1), ferroportin (FPN), ferritin H subunit (FtH) and vinculin. The graphs show densitometric quantification of immunoblot analyses. The values were normalized to vinculin and expressed as a fraction of H69 cells normalized to 1. Mean values ± SEM (n=4), * $p \leq 0.05$, ** $p \leq 0.01$, *** $p \leq 0.001$ vs H69 cell line.

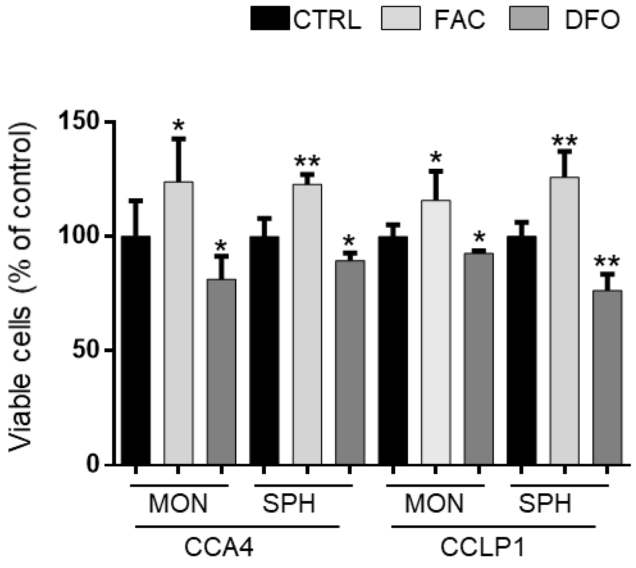

Supplemental Figure 2. Effect of iron on cell viability

CCLP1 and CCA4 human CCA-derived cell lines were cultured as adherent monolayers (MON) or in 3D sphere conditions (SPH) and left untreated (CTRL) or exposed for 18 h to ferric ammonium citrate (FAC) (100 µg/ml) or to Desferioxamine (DFO) (100 µM). Cell viability was evaluated by means of the MTT reduction assay, an indicator of mitochondrial function. Mean values ± SEM (n=3), * $p \leq 0.05$, ** $p \leq 0.01$ vs CTRL MON or SPH for each cell line.

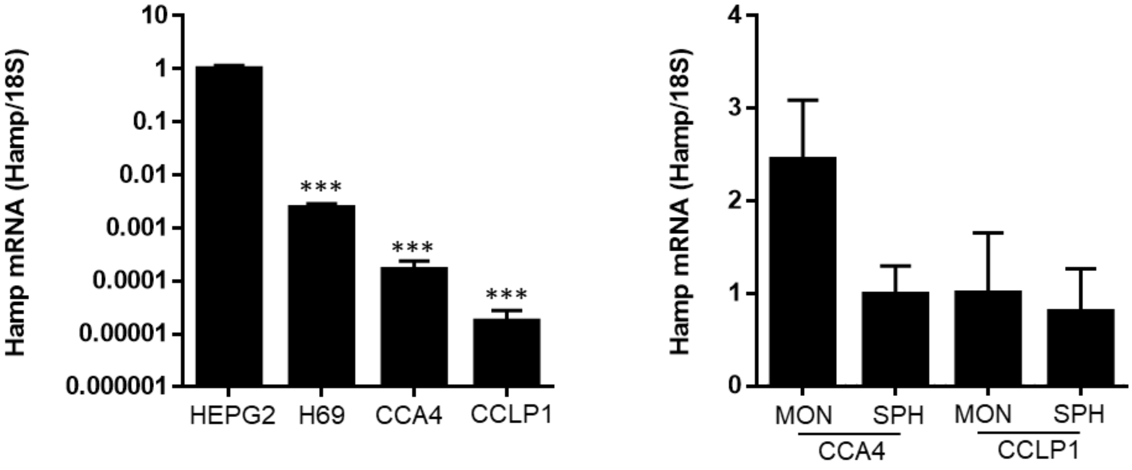

Supplemental Figure 3. Hepcidin expression in different hepatic cell lines

Left: human hepatocarcinoma (HepG2), CCA-derived cell lines and normal immortalized cholangiocytes (H69 cells) were cultured as adherent monolayers. HAMP mRNA levels were measured by quantitative RT-PCR. Samples were analyzed in triplicate, normalized to the housekeeping gene 18S and expressed as percentage of HepG2 cells normalized to 1. Mean values ± SEM (n=4), *** $p \leq 0.001$ vs HepG2. Right: CCLP1 and CCA4 human CCA-derived cell lines were cultured as adherent monolayers (MON) or in 3D sphere conditions (SPH). HAMP mRNA levels were measured by quantitative RT-PCR Samples were analyzed in triplicate, normalized to the housekeeping gene 18S and expressed as percentage of respective MON cells normalized to 1. Mean values ± SEM (n=4).

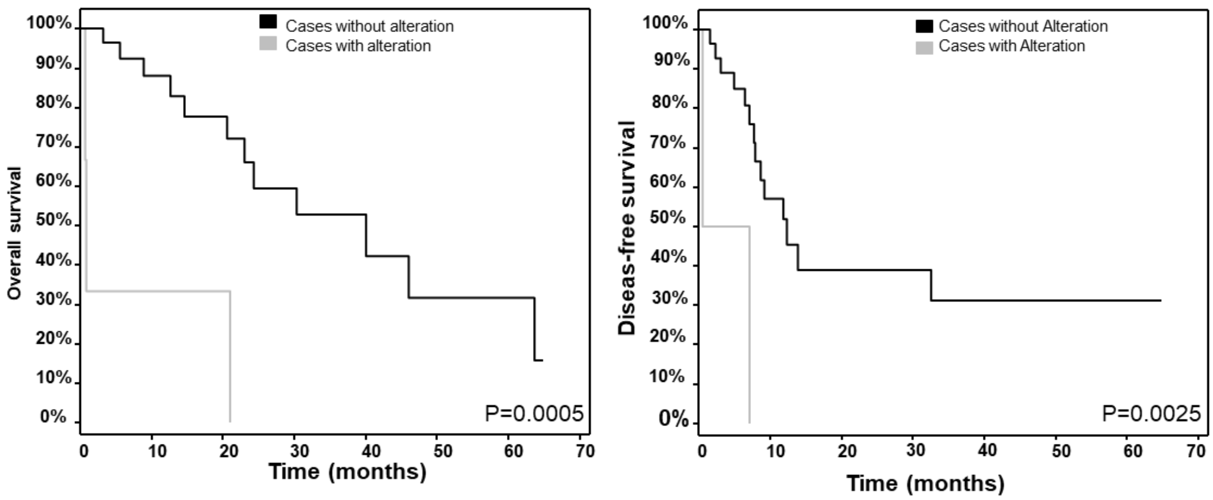

Supplemental Figure 4. Correlation between HAMP expression and overall survival (OS, left) as well as disease-free survival (DFS, right) in the TCGA cohort (n=36 CCA cases). Kaplan-Meier plots show significant differences between cases with HAMP gene alterations (amplification or mRNA upregulation) (gray) versus cases without alterations (black).

k

Supplemental information to Figure 1a

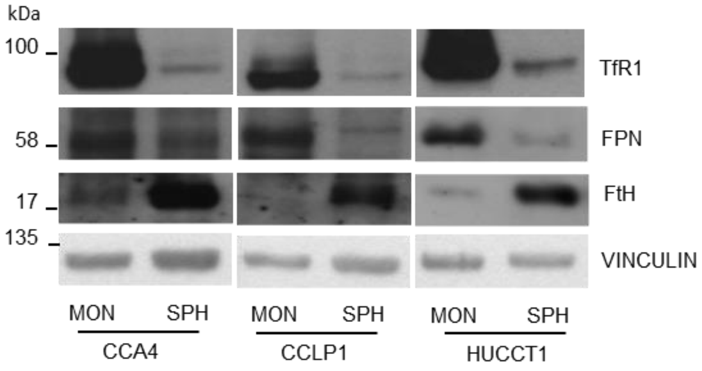

Fig. 1a

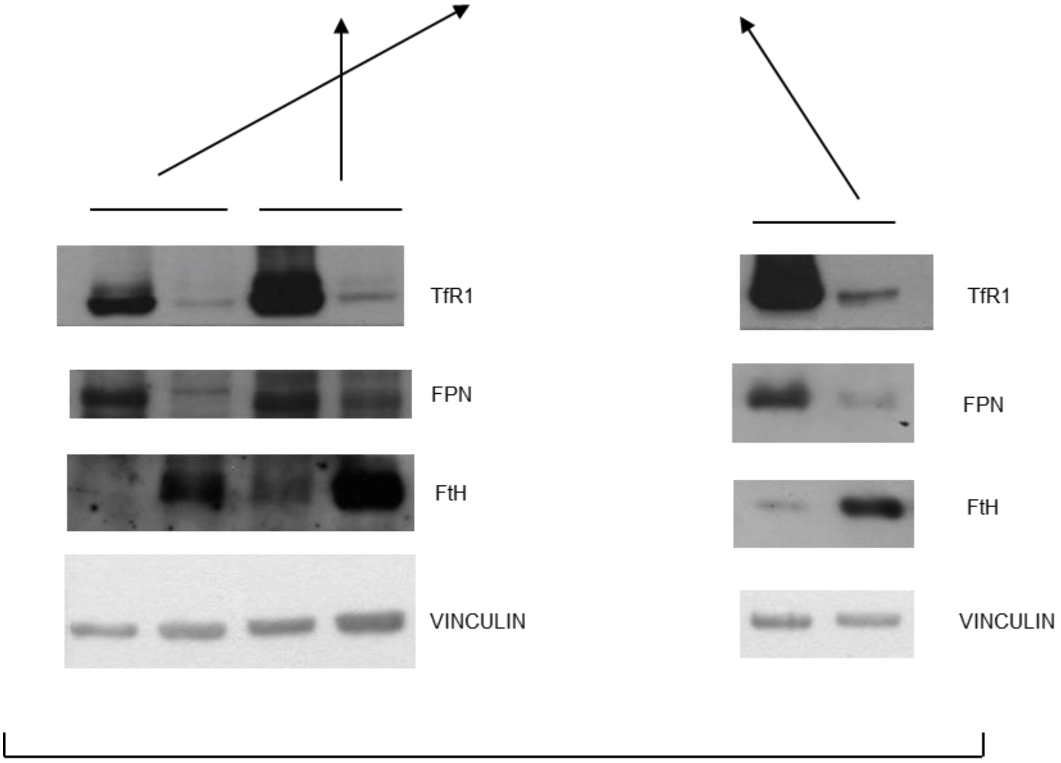

original blots used for
Figure 1a

Supplemental information to Figure 1b

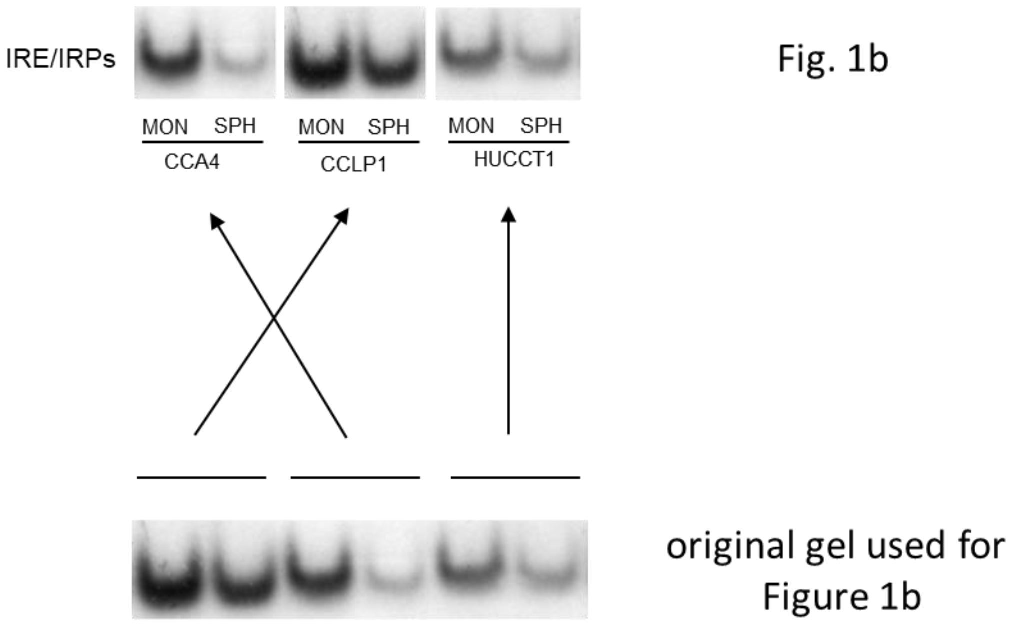

Supplement: Supplementary file 1 — Supplementary Information [file 41598_2017_17804_MOESM1_ESM.pdf]
